# Spatial and temporal distribution of Polycyclic Aromatic Hydrocarbons (PAHs) in sediments from Poyang Lake, China

**DOI:** 10.1371/journal.pone.0205484

**Published:** 2018-10-18

**Authors:** Qianyu Li, Jinglu Wu, Zhonghua Zhao

**Affiliations:** 1 State Key Laboratory of Lake and Environmental Sciences, Nanjing Institute of Geography and Limnology, Chinese Academy of Sciences, Nanjing, China; 2 University of Chinese Academy of Sciences, Beijing, China; National Dong Hwa University, TAIWAN

## Abstract

Concentrations of polycyclic aromatic hydrocarbons (PAHs) were measured in 22 surface sediment samples and an approximately 100-year scale sediment core collected from Poyang Lake. This valuable sediments enable analysis of spatial and temporal distribution patterns of PAH sources, and determine the anthropogenic impacts on Poyang Lake. Total PAH concentrations in the surface sediments ranged from 73.2 to 367.2 ng/g dw, and higher residues were encountered in regions with high-density populations and intensive human activities. Total PAH concentrations in the sediment core ranged from 42.0 to 334.0 ng/g dw and were grouped in two clusters (pre-1990s and post-1990s to the present). PAH concentrations in sediments changed both temporally and spatially, suggesting a difference in PAH sources. Before the 1990s, major PAH sources in the sediment core were from coal, wood and grass combustion. This finding naturally agrees with open lake conditions on a spatial scale, which were related to agricultural activities. Petroleum combustion from industrialization and urbanization has become the predominant PAH source in the sediment core from the 1990s to the present and corresponds to sources observed in the southwestern lake near the relatively developed Nanchang City. In the northern lake leading to the Yangtze River, certain petroleum-related contaminants from shipping have become the main PAH sources. The different PAH sources observed in sediments generally reflect the degree of socio-economic development in the Poyang Lake valley, which is consistent with the local written records, indirectly validating the connection of sediment PAH records to the history of human activities in and around Poyang Lake.

## Introduction

Polycyclic aromatic hydrocarbons (PAHs), which are composed of two or more fused aromatic rings of carbon and hydrogen atoms, are included in lists of hazardous substances and belong to one of the most common families of micro-organic pollutants [1,2]. With population growth and rapid economic increases, the input of PAHs has increased extensively in the 20^th^ century; thus, 16 PAHs are established as priority control pollutants by the U.S. Environmental Protection Agency (USEPA), seven of which are potentially carcinogenic and mutagenic to humans [3,4]. The largest portion of the PAH burden in the environment is derived from the combustion of recent and fossil organic matter (pyrolytic origin), which is generally linked to anthropogenic activity (e.g., coal and wood combustion, petrol and diesel oil combustion, and industrial processes) [5]. Non-anthropogenic sources, such as slow maturation of organic matter under geochemical gradient conditions (of petrogenic origin), short-term diagenetic degradation of biogenic precursors (diagenesis), or degradation of organic matter are comparatively less abundant [6–8]. When PAHs enter the aquatic environment, they can adhere tightly from water to sediments owing to their high hydrophobicity and weak degradation [9,10]. This adherence tendency is especially true of high molecular weight (HMW) PAHs. Although low molecular weight (LMW) PAHs may be degraded during the processing of reaching deep waters and marine sediments with aging [11,12], this processing is comparatively short in shallow freshwater, and the final concentrations of LMW PAHs in sediments are little affected by their degradation [2,13]. Thus, sediments, act as a pollutant sink, a carrier and a secondary source of pollutants that are resuspended in water via physicochemical and biological processes [14,15] and have been identified as an important destination of PAHs in shallow freshwater ecosystems [16].

Poyang Lake, the largest freshwater lake in China, with a maximum water surface area of 4070 km^2^, is located in the northern part of Jiangxi Province and the southern bank of the Yangtze River [17]. It retains water from five rivers (including the Xiushui, Ganjiang, Fuhe, Xinjiang and Raohe) and empties into the Yangtze River through the only outlet in the Hukou Region [18]. Poyang Lake is divided into two parts by the Songmenshan Mountain [19], and the northern part (from the Songmenshan Mountain to Hukou) is a narrow water channel linking the lake and the Yangtze River [20], which has played a crucial role in shipping from Poyang Lake since ancient times. The open lake, at the southern part of Songmenshan Mountain, contributes major economic and social functions to the Jiangxi Province, such as agricultural water use, resident drinking water use and industrial water use [17] and simultaneously takes in waste water from the lake’s watershed through the five rivers. In recent decades, and with the accelerating development of industrialization and urbanization around Poyang Lake, the deteriorating aquatic environment has been cause for extensive concern. Generally, previous environmental investigations of Poyang Lake have concentrated on the lacustrine trophic state changes [21–23] and heavy metal contamination [24,25]. Some studies have begun to address PAH pollution in surface environment components of Poyang Lake [16,26,27], detecting the current contamination status. In this paper, we provided an overview of the spatial and temporal distributions of PAH accumulation in Poyang Lake. Moreover, we also try to identify some of the possible sources of PAHs to further determine the effects of different human activities on Poyang Lake.

## Materials and methods

### Ethics statement and sampling

The sampling in this study was accepted field assistance from the Poyang Lake Laboratory for Wetland Ecosystem Research, Chinese Academy of Science, and all sample collection sites were approved by the Jiangxi Poyang Lake National Nature Reserve Authority. The field studies did not involve protected or endangered species.

**Fig 1:**
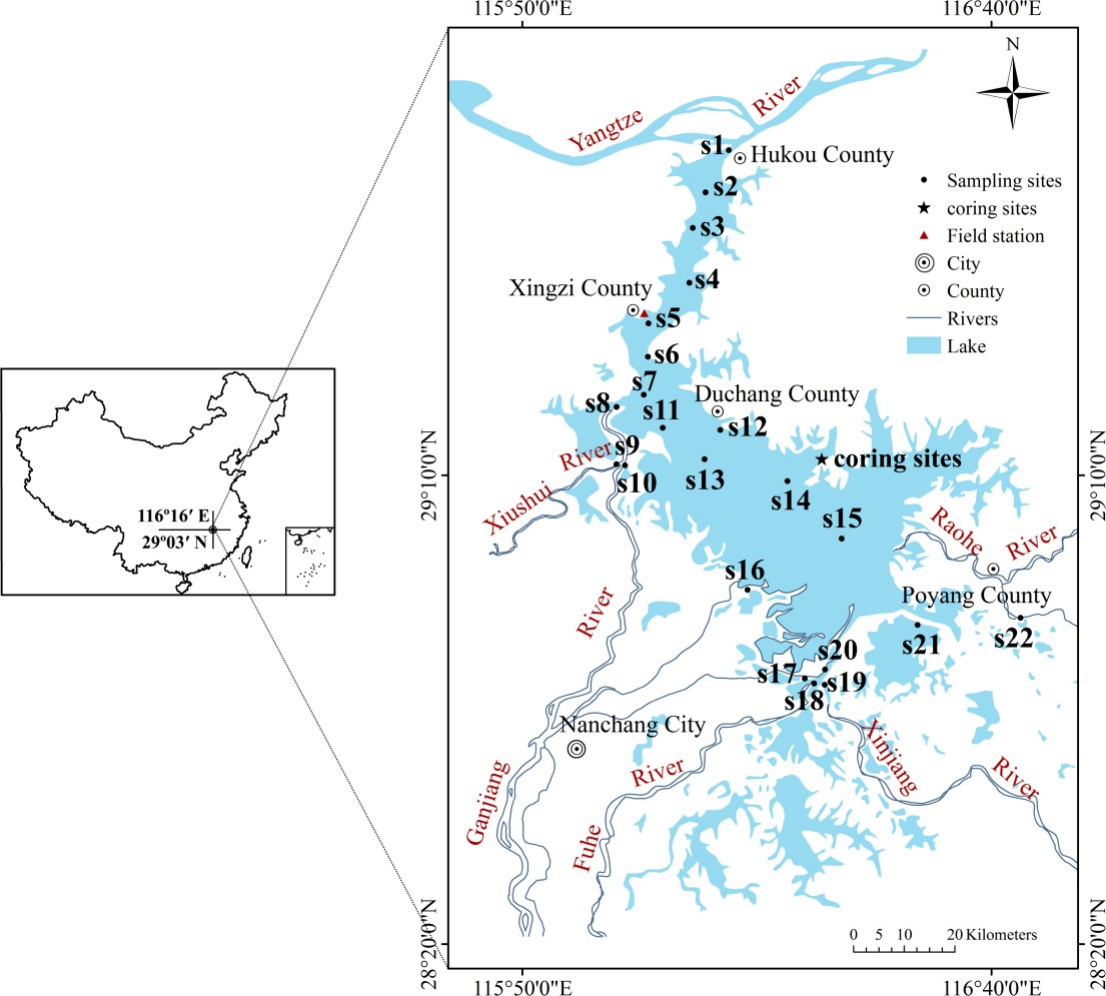
Location and sampling sites of Poyang Lake.

Twenty-two sites (s1-s22) throughout the lake were sampled to analyze the concentrations of PAHs in surface sediments in August 2012. The top 5 cm layer of sediments was scooped using a Peterson grab sampler, homogenized on sites, and then stored in polyethylene bags and kept at 4°C during transportation to the laboratory. When returned to the laboratory, part of samples that do not be contaminated by polyethylene bags were selected. Then, the selected samples were freeze-dried immediately, wrapped up in tinfoil, and stored at -20°C prior to pretreatment process.Two parallel sediment cores (PL1 and PL2 of 54 and 75 cm length, respectively) were collected in November 2011 from Zhouxi Bay, which is situated in eastern Poyang Lake, by using a Piston-percussion corer [28] fitted with 60-mm-internal-diameter Perspex^®^ tubes. Two cores were extruded vertically in the field, and core PL1 was sectioned in 2-cm intervals for PAH analyses, whereas core PL2 were sectioned in 5-cm intervals for ^210^Pb age determination. Each core sample was sealed in a separate polyethylene bag to prevent contamination and was then kept as described for the surface samples. Throughout the survey, a global positioning system (GPS) unit was used to locate the sampling sites shown in Fig 1.

### Chemicals and reagents

Sixteen priority PAHs recommended by the EPA 610 method were purchased from Supelco (USA) for this project. They include naphthalene (NaP), acenaphthylene (Any), acenaphthene (Ana), fluorene (Flu), phenanthrene (Phe), anthracene (Ant), fluoranthene (Flt), pyrene (Pyr), benz[*a*]anthracene (BaA), chrysene (Chr), benzo[*b*]fluoranthene (BbF), benzo[*k*]flouranthene (BkF), benzo[*a*] pyrene (BaP), dibenz[*a*,*h*]anthracene (DahA), benzo[ghi]perylene (BghiP), and indeno[1,2,3-cd]pyrene (IP). The stock standard solutions were dissolved in methanol/dichloromethane (1:1, v/v) and stored in a dark glass container at 4°C. The working solutions were prepared at suitable dilutions daily before use. All of the solvents (dichloromethane, acetone, n-hexane, and methyl alcohol) used for sample processing and analysis were of chromatographic grade (Sinopharm Chemical Reagent Co. Ltd., Shanghai, China). The silica gel (100–200 mesh) and alumina (120–200 mesh) were extracted for 48 h in a Soxhlet apparatus, activated for 12 h at 180°C and 250°C, respectively. They were then deactivated with deionized water at a ratio of 3% (m/m). Deionized water (Millipore Milli-Q System, MA, USA) was used throughout the analysis.

### Sample pretreatment

Freeze-dried samples were ground in an agate mortar to obtain 100 mesh powders for extraction. A total of 5 g of homogenized samples, mixed with 3 g of anhydrous sodium sulfate (baked at 550°C for 6 h), was extracted using an accelerated solvent extraction (ASE) system (Dionex ASE-100, Sunnyvale, CA, USA) as described in Zhao et al. [26]. The extract was further purified with a silica gel-alumina (2:1) power column, and the target n-hexane/dichloromethane elution was then concentrated and treated according to Zhao et al. [29].

### PAH determination

PAH determination was completed using a high-performance liquid chromatography (HPLC) instrument (Agilent 1200 HPLC, USA) equipped with a diode-array detector (DAD) coupled with a series-wound fluorescence detector (FLD). The separation column was a Supelcosil LC-PAHs (ODS, 3.0 mm i.d. × 250 mm length with a particle size: 5 μm from Supelco, USA). Detailed conditions have been described in Zhao et al. [26].

### Quality control and assurance

Peak identification of PAHs was based on both the UV spectra and retention time of the standard components analyzed under the same instrument conditions, whereas the quantification was performed with an external standard method with 6 different concentrations used to generate standard curves between the concentration and peak area for each compound (r = 0.995–0.999). For each set of five samples, a procedural blank and a spike sample consisting of all reagents were run to check for interference and cross contamination. No detected amounts of PAH contaminations were found indicating that contamination was negligible during the transport, storage and analysis of the samples. The limits of detection (LOD) for PAHs were determined as the concentrations of the analytes in a sample that peaked with a signal-to-noise ratio (S/N) of 3, whereas the limits of quantification (LOQ) were determined with a S/N ratio of 10:1. The recoveries for all 16 PAHs were determined as the signal ratio for direct injection of the extract and the working solution prepared in hexane. The recoveries of PAHs ranged from 90% to 116%, and the LOD of the PAHs were in the range of 0.03 to 3.57 ng/g dw.

### Radiometric dating

**Fig 2:**
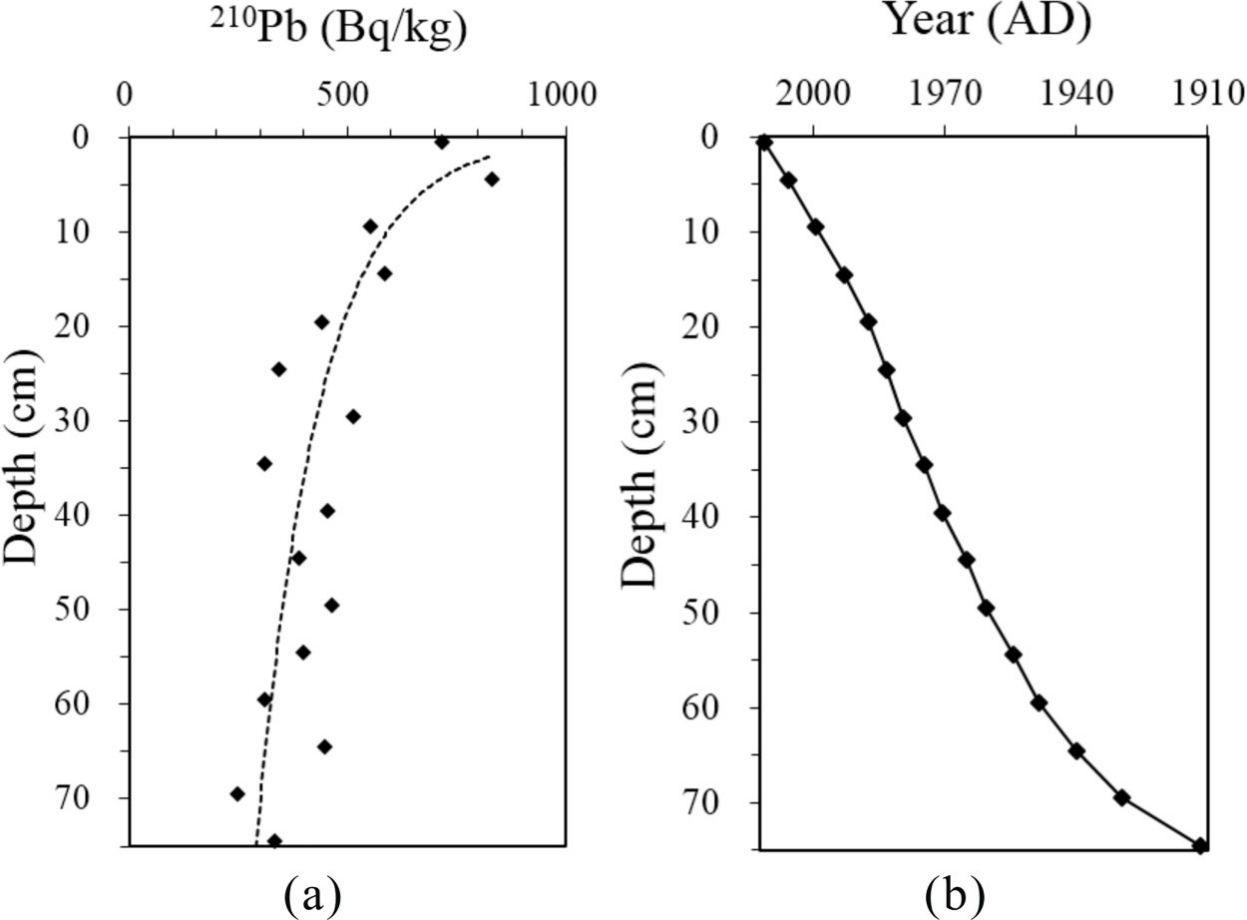
(a) ^210^Pb versus depth in the sediment core and (b) depth-age transformation based on the CRS Pb-model.

Freeze-dried sediments from the core PL2 were analyzed for ^210^Pb by direct gamma spectrometry using an Ortec HPGe GWL series well-type coaxial low-background intrinsic germanium detector. ^210^Pb activity was determined via its gamma emissions at 46.5 keV. The detailed analytical method has been described by Wu et al. [30]. The ^210^Pb activity in core PL2 decreased from 718 Bq/kg at a depth of 0.5 cm to supported levels (246 Bq/kg) at 75 cm and presented a generally exponential decrease with depth. The chronology of the upper 75 cm of the core was determined using a stratigraphic decay profile of ^210^Pb activity and the constant rate of supply (CRS) model of Wang et al. [31] (Fig 2).

### Statistical analysis

PAH concentrations were expressed based on the dry weight (ng/g dry weight, ng/g dw). For samples with concentrations below the LOD or that could not be detected, zero was assigned for statistical purposes. The constrained incremental sum of squares cluster analysis (CONISS) was performed using the program PAST version 3.01 [32] to identify significantly different zones in the sediment core. The principal component analysis with multiple linear regression (PCA/MLR) was conducted with the IBM SPSS Statistics 20.0 for Windows (IBM, Armonk, NY, USA).

## Results

### PAHs in the surface sediments

**Fig 3:**
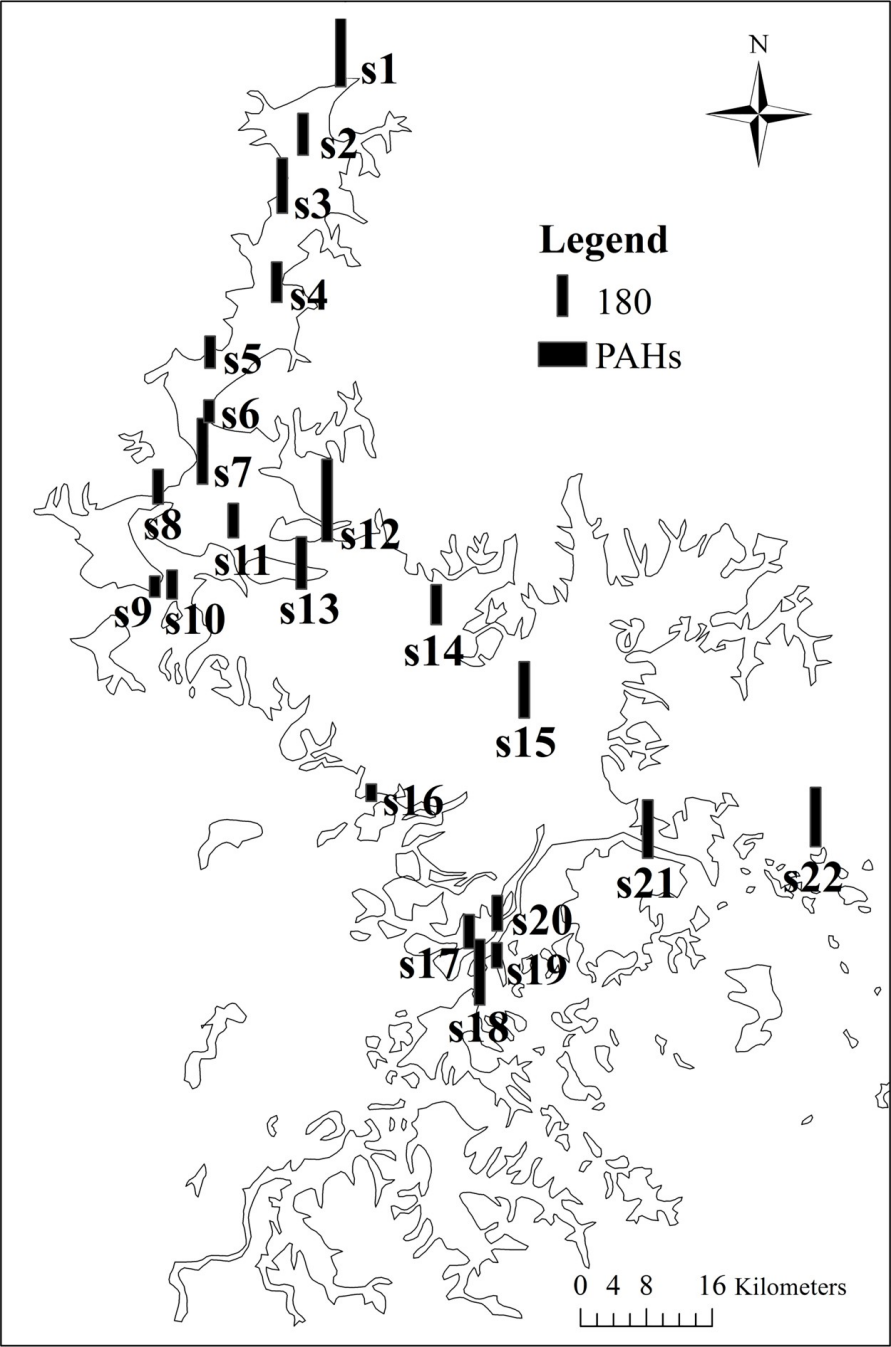
Spatial distribution and concentrations of PAHs in surface sediments from Poyang Lake (the black bar of PAHs in the legend denots 180 ng/g dw).

The concentrations of total PAHs and individual compounds in the surface sediments are illustrated in Table 1. A total of 16 types of PAHs were detected in all samples, and the residual levels ranged from 73.2 ng/g dw to 367.2 ng/g dw (a mean concentration of 192.8±77.6 ng/g dw). Among the PAHs, a 2-ringed PAH (Nap) was observed in the range of 4.4–56.5 ng/g dw (24.9±10.9 ng/g dw) and 3-ringed PAHs (sum of Any, Ace, Flu, Phe and Ant) ranged from 21.4 to 146.8 ng/g dw with a mean value of 70.3±30.8 ng/g dw. For HMW PAHs, the levels of 4-ringed (sum of Flt, Pyr, BaA and Chr), 5-ringed (sum of BbF, BkF, BaP and DahA) and 6-ringed PAHs (sum of BghiP and IP) ranged from 8.2 to 228.3 ng/g dw (53.3±48.6 ng/g dw), 0.1 to 35.8 ng/g dw (33.1±7.1 ng/g dw) and 4.6 to 99.5 ng/g dw (26.7±30.0 ng/g dw), respectively. Flt was the most predominate PAH compound (33.2±35.1 ng/g dw), followed by Phe (29.9±15.0 ng/g dw) and IP (27.3±34.0 ng/g dw), whereas BkF, DahA and BaP were found at the lowest residual levels. In addition, in spatial distributions (Fig 3), higher residual levels of total PAHs were found at site s12 (367.2 ng/g dw), s1 (297.3 ng/g dw), s18 (280.8 ng/g dw) and s22 (254.5 ng/g dw), which were located close to regions with high-density populations and a high rate of human activities. However, lower total PAH concentrations were more observed in the open lake, and the lowest was in site s16 (73.2 ng/g dw).

**Table 1:** Detection values of individual PAH compounds in surface sediments from Poyang Lake.

compounds	Minimum	Maximum	Mean	Standard deviation	Detection frequency
	(ng/g dw)	(ng/g dw)	(ng/g dw)	(ng/g dw)	(%)
Nap	4.4	56.5	24.9	10.9	100.0
Acy	Nd*	52.8	11.0	9.6	95.5
Ace	1.6	60.3	15.8	16.4	100.0
Flu	1.8	29.9	13.3	7.2	100.0
Phe	10.0	72.4	29.9	15.0	100.0
Ant	0.2	12.2	3.9	3.0	100.0
Flt	3.6	125.2	33.2	35.1	100.0
Pyr	4.3	88.3	19.1	18.3	100.0
BaA	Nd*	56.2	10.7	14.4	81.8
Chr	0.2	7.4	2.1	1.8	100.0
BbF	Nd*	28.1	5.7	7.7	63.6
BkF	Nd*	5.7	1.1	1.4	81.8
BaP	0.1	9.5	1.6	1.9	100.0
DahA	Nd*	3.4	1.5	0.8	72.7
BghiP	1.2	24.9	7.8	6.7	100
IP	Nd*	98.4	27.3	34.0	72.7
∑PAHs	73.2	367.2	192.8	77.6	100.0

* Nd: the concentrations of the compounds less than the LODs were defined as not detectable.

### PAHs in the sediment core

**Fig 4:**
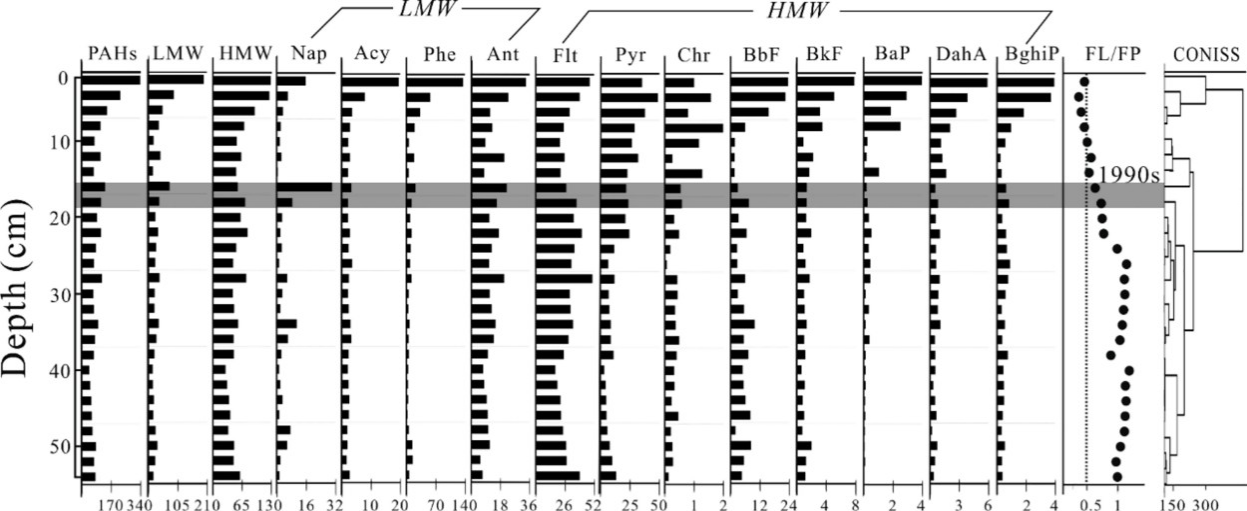
Concentrations of PAHs (ng/g dw) and diagnostic ratios of FL/FP (Flt/(Flt+Pyr)) in the sediment core from Poyang Lake.

A total of 12 types of PAHs were detected from the original 16 compounds (Ace, Flu, BaA and IP were absent). The total PAHs in the sediment core ranged from 42.0 ng/g dw to 334.0 ng/g dw, with a mean concentration of 96.6±59.7 ng/g dw. LMW PAHs (sum of 2- and 3-ringed PAHs) were found at values of 14.2–210.4 ng/g dw (38.6±39.2 ng/g dw), in which Phe was the most abundant compound (16.7±27.1 ng/g dw). The residual levels of HMW PAHs (sum of 4-, 5- and 6-ringed PAHs) were detected in the range of 27.8–123.6 ng/g dw (with a mean concentration of 58.0±23.7 ng/g dw). Flt was also a predominant compound (30.9±7.7 ng/g dw) followed by Pry (15.8±11.9 ng/g dw). In general, total PAH concentrations showed an upward trend in the sediment core and reached a peak at the top of the section (Fig 4). A cluster analysis (constrained incremental sum of squares cluster analysis, CONISS) revealed that the vertical profile of total PAHs could be divided into two stages: 1) prior to the 1990s and 2) 1990s to the present. Prior to the 1990s, total PAH concentrations were low and fluctuated between 50 ng/g dw and 100 ng/g dw. Thereafter, total PAH concentrations fell slightly and then increased rapidly to the present. The vertical profiles of LMW and HMW PAHs were similar to that of total PAHs.

## Discussion

**Fig 5:**
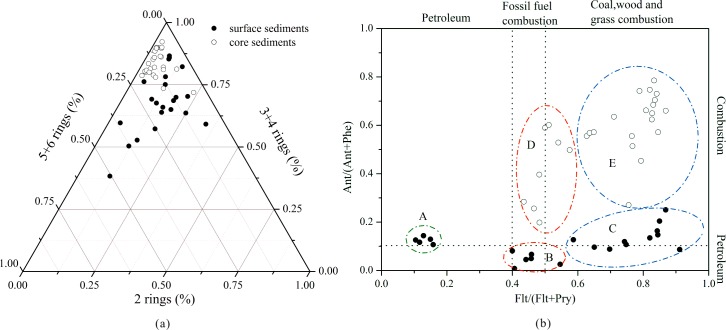
(a) PAH compositions and (b) the diagnostic ratio of Ant/(Ant+Phe) vs. Flt/(Flt+Pyr) in sediments from Poyang Lake.

PAHs emitted from different sources exhibit different molecular compositions; therefore, analysis of PAH compositions helps elucidate the PAH sources [2]. The discharge of petrogenic/petroleum-related materials is abundant with LMW PAHs, especially 2-ringed PAHs. The composition of combustion-derived PAHs is dependent on the source temperature; that is, at lower temperatures, such as that used for the combustion of coal and wood, 3- and 4-ringed PAHs compounds are predominant [33], whereas at higher temperatures, such as those for the combustion of petroleum fuels, 5- and 6-ringed PAHs compounds are prominent [34]. As seen in Fig 5A, the compositions of PAHs were dominated by 3- and 4-ringed PAHs regardless of whether they were in the surface sediments or in the core sediments (67.8%±12.2% for the surface sediments and 83.5%±5.3% for the core sediments), but there were still some differences between the surface and core sediments. The composition of PAHs in the core sediments was more centralized at the top of the ternary chart and the axis of 5+6 rings compared with that of the surface sediments, thus suggesting that the main PAH sources in the core sediments were due to low-temperature combustion mixed with high-temperature combustion. In addition, the compositions of PAHs in the surface sediments were relatively scattered, thus suggesting that the origin of PAHs in the surface sediments may be more complicated.

To assess PAH sources more accurately, diagnostic ratios of selective individual compounds are considered to be good indicators, and several markers are widely used to determine the origin of PAHs presented in different environments [35,36]. Ant/(Ant+Phe) is frequently used to distinguish between combustion and petrogenic/petroleum-related sources. A ratio of <0.1 indicates petrogenic/petroleum-related sources, and a ratio of >0.1 indicates a dominance of combustion [37]. In addition, the ratio of Flt/(Flt+Pyr) is also used because the Flt and Pyr isomer pair degrade photolytically at comparable rates [38,39]. Yunker and Macdonald [38] have suggested that a Flt/(Flt+Pyr) ratio of <0.4 indicates petroleum-related residues; a Flt/(Flt+Pyr) ratio in the range of 0.4–0.5 indicates fossil fuel combustion, such as the burning of industrial coal and petroleum fuels; and a Flt/(Flt+Pyr) ratio of >0.5 indicates the combustion of domestic coal, grass and wood.

According to the combined patterns of these two diagnostic ratios in the sediments, surface sediments were divided into three groups (A, B and C), whereas core sediments were divided into two groups (D and E) (Fig 5B). Group A includes s1-s5, of which the Flt/(Flt+Pyr) ratios were all less than 0.4 and the Ant/(Ant+Phe) ratios were approximately 0.1, reflecting the typical contribution of petroleum-related sources. All of these sample sites were located in the narrow water channel in northern Poyang Lake, which is the only outlet connecting the lake with the Yangtze River (Fig 3). This water channel has palyed a pivotal role in heavy water transportation since ancient times and is probably associated with the production of certain petroleum-related materials or oil leakage in this region [40]. Group B includes s16-s21, most of which are located in southwestern Poyang Lake, near Nanchang City, which is the largest city with a high level of industrialization and urbanization around Poyang Lake. The ratios of Ant/(Ant+Phe)<0.1 and 0.4<Flt/(Flt+Pyr)<0.5 in this region suggested mixed sources of petroleum-related pollution and fossil fuel combustion. Group C included the rest of the sample sites located in the open lake, which were partly effected by highly productive agricultural land and a rural population. The Ant/(Ant+Phe) and Flt/(Flt+Pyr) ratios were more than 0.1 and 0.5, respectively, indicating a predominant source of coal, grass and wood combustion. Fossil fuel combustion at high temperatures is mainly from industrial and urban development processes, whereas coal, wood and grass combustion at lower temperatures are primarily produced by agricultural activities [41]. Therefore, PAH sources in these three areas were influenced by shipping, industrial activities and agricultural activities. In addition to these spatial differences, the diagnostic ratios in the sediment core varied temporally, again indicating a change in the sources of PAHs. Sediments from the base of the core to 16 cm (prior to the 1990s) made up group D, and the ratios of Ant/(Ant+Phe)>0.1 and Flt/(Flt+Pyr)>0.5 were attributed to the combustion of grass, wood and coal. This result was similar to that for group C, speculating that the PAHs were probably generated by agricultual activities before the 1990s. Sediments above 16 cm (after the 1990s) were made up of group E which had Ant/(Ant+Phe) ratios of more than 0.1 and Flt/(Flt+Pyr) ratios primarily between 0.4 and 0.5, except that the sediments from 12 to 16 cm were in a phase of transition from lower sediments to upper sediments with Flt/(Flt+Pyr) ratios approaching 0.5. Consequently, PAH sources in group E varied in origination from grass, wood, and coal combustion to fossil fuel combustion, thus suggesting that the main human activities have probably shifted from agriculture to industry around Poyang Lake since the 1990s.

**Fig 6:**
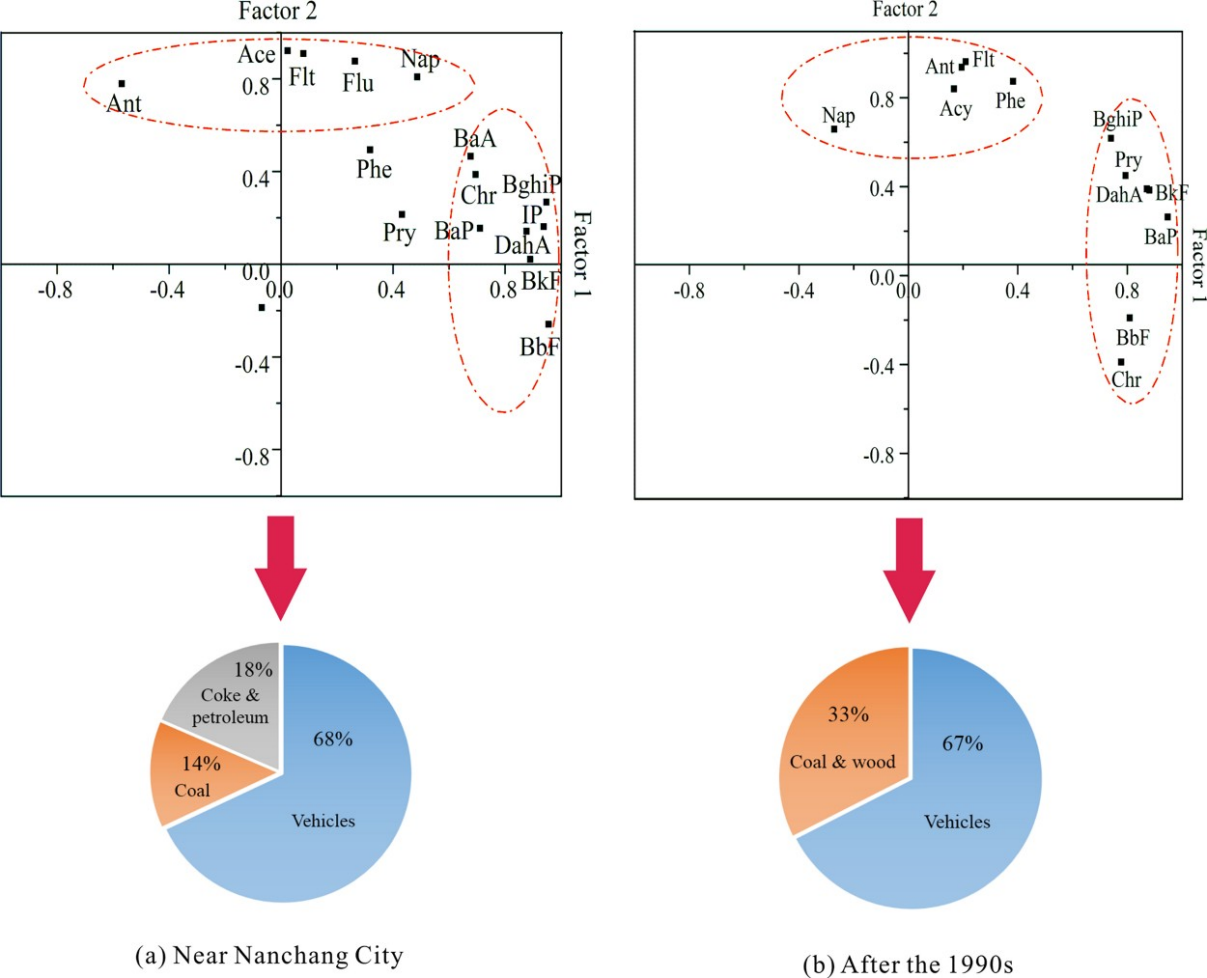
Loading of the principal component for PAH and contributions of the major driving force of PAH sources in sediments (a) near Nanchang City and (b) after the 1990s.

To try to further explore the major driving force and possible contributions of PAH sources with the impacts of intensive human activities, a principal component analysis with multiple linear regression (PCA/MLR) was used to analyze sediments near Nanchang City and after the 1990s (Fig 6). Three principal components (PCs) were extracted and rotated in sediments near Nanchang City and accounted for 90.1% of the total variance. The first component (PC1), second component (PC2) and third component (PC3) represented 39.5%, 28.1% and 22.5%, respectively. PC1 had strong positive loadings for HMW PAHs, such as InP, BbF, BkF, BghiP and DahA and had moderate positive loadings for BaP, BaA and Chr. Among these PAHs, InP, BbF and BkF were the tracers of diesel-powered vehicles [42,43], whereas DahA, BghiP and BaP were related to gasoline-powered vehicles [44–46]. Thus, PC1 reflected the petroleum combustion from vehicles. Vehicle energy consumption has become the main factor that influences the petroleum combustion in relatively developed cities [47]. According to the statistics from the Xinhua News Agency [48], given the rapid development of the urbanization process, the number of vehicles in Nanchang City has increased tenfold over the past decades. PC2 was highly weighted by Flu, Ace, Ant and Nap. Flu, Ace and Nap have been reported to be the dominant PAHs in a coke oven signature, and Nap is also related to petroleum-derived pollution [42,49,50]. Therefore, PC 2 was indicative of mixed sources of coke combustion and petroleum. Coke production and petroleum hydrocarbon production are primarily from low-end industries, hence, the proliferation of low-end industries in Nanchang City may be the driving force of PC2. PC3 was dominated by Phe, Pry and BaA, which have been identified as markers of domestic coal combustion [51,52]. For a quantitative analysis of the relative contributions of various sources, a multiple linear regression (MLR) was conducted. PCA factor scores PC1, PC2 and PC3 were regressed against the standard normalized deviate of 16 PAH concentrations. The results showed that the percentage of total contribution was 68% for vehicles, 18% for coking and petroleum pollution, and only 15% for coal combustion. That is, in the process of industrialization and urbanization, pollution from PAHs was predominantly from vehicles and low-end industries.

Two PCs were extracted in the core sediments after the 1990s, and the cumulative variance accounted for 83.7% of the total variance, of which PC1 and PC2 was responsible for 43.4% and 40.3%, respectively. PC1 was characterized by high loadings of BbF, BkF, BaP, DahA and BghiP. These compounds are considered good markers of vehicles engine emissions [42,43,46]. PC2 was predominately loaded on Ant, Phe and Flt, and is regarded as a significant marker of domestic coal and wood combustion [51,52]. The burning of wood and domestic coal for indoor cooking and heating, and the burning of straw for material reduction and fertilizing are typically agricultural activities. MLR was used to analyze the average contributions of these two sources. Petroleum combustion from vehicles was the main driving force (67% of the total contribution), whereas coal and wood combustion accounted for only 34% (Fig 6B). Hence, urban development has been the major trend around Poyang Lake after the 1990s, but rural production has also had an influence, thus reflecting that agricultural activities dominated prior to the 1990s.

Overall, PAH sources reflected the type and intensity of human activities in and around Poyang Lake. The sediment core recorded that the origin of PAHs was primarily from coal, wood and grass combustion at low temperatures prior to the 1990s, indirectly indicating that agricultural production practices were predominant during this period. This result corresponded to the surface sediment records in the open lake on a spatial scale. In contrast, after the 1990s, petroleum fuel combustion at high temperatures from industries and vehicles was the principal PAH source, a result consistent with the result of surface sediments near Nanchang City, thus suggesting a relatively modernized economic structure. Nanchang City, which is the center of Poyang Lake Ecological Economic Zone, is a megacity of over 5 million people, in which most of gross domestic product (GDP) is derived from industry [53]. Furthermore, the increasing number of vehicles in Nanchang City promoted the petroleum consumption [47]. Meanwhile, the proportion of agriculture in the Poyang Lake Ecological Economic Zone has experienced a sharp decline since the 1990s, whereas industry rose by 14.4% from 1990 to 2012. The energy consumption structure has been in step with the economic structure; that is, coal consumption has decreased while petroleum consumption has increased during the past two decades [54]. Therefore, the concentration and composition of PAHs observed in sediments from Poyang Lake generally reveal the degree of socio-economic development in the basin and probably further imply that sediment PAHs are a good proxy for anthropogenic impacts on Poyang Lake, which is also evidenced by documentary records.

## Conclusions

The spatial and temporal distributions of sediment PAHs from Poyang Lake are mainly influenced by human activities. During the past 100 years, Poyang Lake has undergone a 2-stage history of contrasting PAH source changes in response to human activities. The origin of PAHs in core sediments deposited prior to the 1990s was dominated by coal, wood and grass combustion as a result of agricultural practices, as evidenced by the PAH sources in surface sediments from the open lake in spatial distribution. With the accelerating development of industrialization and urbanization, petroleum fuel combustion generated by intensified industrial and urban activities has become the predominant PAH source since the 1990s, as indicated by surface sediments in southwestern Poyang Lake near Nanchang City. Some petroleum-related contamination in northern Poyang Lake, which has the only outlet joining the Yangtze River, is probably linked to busy water transportation. The distribution of sediment PAHs corresponded well with the data of local economic structure and energy consumption; hence, sediment PAHs from Poyang Lake provides specific information regarding anthropogenic impacts.
